# A Cyp51B Mutation Contributes to Azole Resistance in *Aspergillus fumigatus*

**DOI:** 10.3390/jof6040315

**Published:** 2020-11-26

**Authors:** Irene Gonzalez-Jimenez, Jose Lucio, Jorge Amich, Isabel Cuesta, Rafael Sanchez Arroyo, Laura Alcazar-Fuoli, Emilia Mellado

**Affiliations:** 1 Mycology Reference Laboratory, National Centre for Microbiology, Instituto de Salud Carlos III (ISCIII), Majadahonda, 28222 Madrid, Spain; irene.gonzalez@isciii.es (I.G.-J.); jose.lucio@isciii.es (J.L.); lalcazar@isciii.es (L.A.-F.); 2 Manchester Fungal Infection Group (MFIG), Division of Infection, Immunity and Respiratory Medicine, University of Manchester, Manchester M13 9PL, UK; jorge.amichelias@manchester.ac.uk; 3 Bioinformatics Unit, Common Scientific Technical Units, Instituto de Salud Carlos III (ISCIII), Majadahonda, 28222 Madrid, Spain; icuesta@isciii.es; 4 Microbiology Laboratory, Hospital de Ávila, 05004 Ávila, Spain; rsancheza@saludcastillayleon.es; 5 Spanish Network for Research in Infectious Diseases (REIPI RD16/CIII/0004/0003), Instituto de Salud Carlos III (ISCIII), Majadahonda, 28222 Madrid, Spain

**Keywords:** azole resistance, *Aspergillus fumigatus*, Cyp51B, Cyp51A, Hmg1

## Abstract

The emergence and spread of *Aspergillus fumigatus* azole resistance has been acknowledged worldwide. The main problem of azole resistance is the limited therapeutic options for patients suffering aspergillosis. Azole resistance mechanisms have been mostly linked to the enzyme Cyp51A, a target of azole drugs, with a wide variety of modifications responsible for the different resistance mechanisms described to date. However, there are increasing reports of *A. fumigatus* strains showing azole resistance without Cyp51A modifications, and thus, novel resistance mechanisms are being explored. Here, we characterized two isogenic *A. fumigatus* clinical strains isolated two years apart from the same patient. Both strains were resistant to clinical azoles but showed different azole resistance mechanisms. One strain (CM8940) harbored a previously described G54A mutation in Cyp51A while the other strain (CM9640) had a novel G457S mutation in Cyp51B, the other target of azoles. In addition, this second strain had a F390L mutation in Hmg1. CM9640 showed higher levels of gene expression of *cyp51A*, *cyp51B* and *hmg1* than the CM8940 strain. The role of the novel mutation found in Cyp51B together with the contribution of a mutation in Hmg1 in azole resistance is discussed.

## 1. Introduction

*Aspergillus fumigatus* is an opportunistic human fungal pathogen that affects immunocompromised patients causing a wide range of pathologies called aspergillosis [1]. Among them, invasive aspergillosis (IA) is the most lethal fungal infection caused by this mold, affecting over 300,000 people a year, with a mortality rate ranging from 30–80% [2].

The first-line treatment for aspergillosis is based on the use of azoles, a class of antifungal drugs that target the 14-α sterol demethylase, a key enzyme in the ergosterol biosynthesis pathway [3]. Within the azole drugs family, only four triazole agents (voriconazole, itraconazole, isavuconazole and posaconazole) have been approved for clinical use in the treatment and prophylaxis of aspergillosis [4]. *A. fumigatus* has a complex ergosterol biosynthesis pathway with several gene duplications, as in the genes encoding the two 14-α sterol demethylase paralogous enzymes, Cyp51A and Cyp51B [5,6]. Both enzymes are targets of the azole drugs, and together, are known to be essential for ergosterol production and fungal growth [7,8]. Also, both deletion mutants (*cyp51A* and *cyp51B)* have been described as more susceptible to azole antifungals [7,8].

In the last decade, the number of clinical *A. fumigatus* isolates that are resistant to triazole drugs has increased, causing considerable problems in the treatment of aspergillosis patients as their therapeutic options are being reduced [4]. To date, the majority of the *A. fumigatus* azole resistance mechanisms are linked to mutations in the *cyp51A* gene, which cause amino acid changes in the protein sequence or overexpression of the gene. These modifications can be single non-synonymous point mutations in the coding sequence of the gene (G54, G138, P216, M220 and G448), tandem repeat (TR) insertions in the promoter of *cyp51A* (TR53) or the combination of both (TR34/L98H, TR34/R65K/L98H and TR46/Y121F/T289A) [9,10].

More recently, the reports of *A. fumigatus* azole resistant isolates, without Cyp51A modifications, indicate that there are other novel mechanisms mediating resistance to azole drugs [11,12]. These studies have reported roles in azole resistance of other genes from the ergosterol biosynthesis pathway, including *hmg1* [13,14,15,16,17,18,19], *erg6* [15,17], some transcriptional factors [20,21,22], *hapE* [23] or overexpression of drug efflux pumps [24,25].

Fungal cytochrome P450 14-α sterol demethylases (Cyp51s) are required for fungal ergosterol biosynthesis and are the target for azole antifungal compounds [26]. In fungi the number of 14-α sterol demethylase enzymes is variable. Yeast (all *Candida* spp.), basidiomycetes (as *Cryptococcus* spp. and *Ustilago maydis*), other filamentous fungi including *Scedosporium* spp. and most plant pathogens have only one 14-α sterol demethylase enzyme named Erg11/Cyp51 [27,28,29]. Most *Aspergillus* spp., *Penicillium* spp. and *Mucorales* spp. have two paralogues (Cyp51A and Cyp51B) and a few species including *A. flavus, A. oryzae* and the genus *Fusarium* have three Cyp51 enzymes (Cyp51A, Cyp51B and Cyp51C) [30,31]. Phylogenetically, Cyp51B enzymes and all Cyp51s of those species with only one enzyme form a clear subgroup while the Cyp51A proteins are placed in a different phylogenetic subgroup [32].

In *A. fumigatus*, Cyp51A and Cyp51B, encoded by *cyp51A* and *cyp51B* genes, share 59.4% of their sequence identity [5]. Despite Cyp51B functioning as a Cyp51A alternative [7,8], the implication of Cyp51B in clinical azole resistance has only been reported in one study [11]. Buied et al. identified over-expression levels of *cyp51B*, constitutive and azole inducible, in a couple of clinical azole resistant isolates with a wild-type *cyp51A*, suggesting a possible link to azole resistance [11]. However, the mechanism leading to Cyp51B increased expression has yet to be characterized. In addition, a few studies have reported mutations and polymorphisms in the *cyp51B* sequence in azole-resistant and also in azole-susceptible strains [33,34,35]. Even if these last mutations are not implicated in azole resistance, the fact that the *cyp51B* gene presents substantial variability sets it as a possible candidate to explain non-*cyp51A* associated resistance mechanisms.

Indeed, in some fungal pathogens, azole resistance mechanisms have been related to modifications in the homologous Cyp51B [36]. In previous studies, the Cyp51B modifications are usually ignored, although they could be responsible for azole resistance in *A. fumigatus* in strains without a known Cyp51A azole resistance mechanism. In this study, we identify and characterize a clinical *A. fumigatus* panazole resistant isolate with a novel G457S substitution in Cyp51B located in an important domain of the enzyme that seems to be responsible for azole resistance. Additionally, this isolate has a F390L mutation in Hmg1 which could contribute to the high azole resistance phenotype of the strain. We further discuss the possible implications of the two mutations combined (Cyp51B and Hmg1).

## 2. Materials and Methods

### 2.1. A. Fumigatus Strains

In this study we analyze two *A. fumigatus* strains (CM8940 and CM9640) that were isolated from respiratory samples of a patient suffering from a chronic pulmonary pathology with long-term azole therapy at Hospital de Ávila, in Spain. The first isolate (CM8940) was obtained in February 2017 and it was identified as an azole resistant *A. fumigatus*. Two years later, in June 2019 a second isolate (CM9640) also identified as an azole resistant *A. fumigatus* was collected. Both strains were confirmed as *A. fumigatus* by PCR amplification and sequencing of ITS1-5.8S-ITS2 regions and a portion of the β–tubulin gene [37]. Previously, in September 2016 an *A. lentulus* (CM8693) isolate was identified in a sputum sample from the same patient.

### 2.2. Clinical Antifungal Drugs Susceptibility Testing

Antifungal susceptibility testing (AFST) was performed following the European Committee on Antimicrobial Susceptibility Testing (EUCAST) broth microdilution reference method 9.3.1 [38,39]. Antifungals used were amphotericin B (Sigma-Aldrich Química, Madrid, Spain), itraconazole (Janssen Pharmaceutica, Madrid, Spain), voriconazole (Pfizer SA, Madrid, Spain), posaconazole (Schering-Plough Research Institute, Kenilworth, NJ, USA) and isavuconazole (Basilea Pharmaceutica, Basel, Switzerland (tested from January 2017)). The final concentrations tested ranged from 0.03 to 16 mg/L for amphotericin B and 0.015 to 8 mg/L for the four azoles. *A. flavus* ATCC 204304 and *A. fumigatus* ATCC 204305 were used as quality control strains in all tests performed. Minimal inhibitory concentrations (MICs) were visually read after 24 and 48 h of incubation at 37 °C in a humid atmosphere. MICs were performed at least twice for each isolate. Clinical breakpoints for interpreting AFST results established by EUCAST [40] were used for classifying the *A. fumigatus* strains as susceptible or resistant.

### 2.3. Environmental Drugs Susceptibility Testing

AFST with 14-α demethylation inhibiting fungicides (DMIs) was performed following the EUCAST methodology as described before. Environmental DMIs tested for microdilution assay were two imidazole drugs, imazalil and prochloraz; and five triazole drugs, tebuconazole, epoxiconazole, difenoconazole, bromuconazole and metconazole, all of them purchased at Sigma-Aldrich Química, Madrid, Spain. The final concentrations of each azole drug tested ranged from 0.06 to 32 mg/L. MICs were visually read as described before. Clinical breakpoints for DMIs have not been standardized yet so wild-type strain MICs were considered as susceptible.

### 2.4. Cyp51A, Cyp51B and Hmg1 Amplification, PCR Conditions and Sequencing

For DNA extraction, conidia from each strain were cultured in glucose-yeast extract-peptone (GYEP) liquid medium (0.3% yeast extract, 1% peptone; Difco, Soria Melguizo, Madrid, Spain) with 2% glucose (Sigma-Aldrich Química, Madrid, Spain) for 24 h at 37 °C. After mechanical disruption of the mycelium by vortex-mixing with glass beads, genomic DNA of isolates was extracted using the phenol-chloroform method [41].

The full coding sequences of the *cyp51A* and *cyp51B* genes, including their promoters, and the *hmg1* gene were amplified and sequenced. To exclude the possibility that any change identified in the sequences was due to PCR-induced errors, each isolate was independently analyzed twice. Primers used to amplify the sequence of the genes included in this study are listened in Table S1.

PCR reaction mixtures contained 0.5 μM of each primer, 0.2 μM of deoxynucleoside triphosphate (Roche, Madrid, Spain), 5 μL of PCR 10x buffer, 2 mM of MgCl_2_, DMSO 5.2%, 2.5 U of Taq DNA polymerase (Applied Biosystems, California, USA), and 100–200 ng of DNA in a final volume of 50 μL. A DNA 1-kb molecular ladder (Promega, Madrid, Spain) was used for all electrophoresis analyses. The samples were amplified in a GeneAmp PCR System 9700 (Applied Biosystems, California, USA). The parameters used were 1 cycle of 5 min at 94 °C and then 35 cycles of 30 s at 94 °C, 45 s at 56 °C for *cyp51A* promoter and *hmg1* P7 and P8, 58 °C for *cyp51A*, *cyp51B* and *cyp51B* promoter, 60 °C for *hmg1* P1 and P2, P5 and P6, P9 and P10, 55 °C for *hmg1* P3 and P4, and 2 min at 72 °C, followed by a 1 final cycle of 5 min at 72 °C. The amplified products were purified using IllustraExoprostar 1 –step (GE Helthcare Life Science, Buckinghamshire, UK) and both strands were sequenced with the Big-Dye terminator cycle sequencing kit (Applied Biosystems, Foster City, California, USA) following manufacturer’s instructions, using the primers listed in Table S1. All gene sequences were edited and assembled using Lasergene software package (DNAStar Inc., Madison, WI, USA).

### 2.5. Strains Genotyping

The two *A. fumigatus* strains included in this study (CM8940 and CM9640) were genotyped following the previously described typing method TRESPERg [42,43]. Four markers were used: (i) Afu2g05150 encoding an MP-2 antigenic galactomannan protein (MP2), (ii) Afu6g14090 encoding hypothetical protein with a CFEM domain (CFEM), (iii) Afu3g08990 encoding a cell surface protein A (CSP) and (iv) Afu1g07140 (ERG), which encodes a putative C-24(28) sterol reductase. The combination of the genotypes obtained with each marker has a discriminatory value (D) of 0.9972 using the Simpson index.

### 2.6. RNA Isolation and Reverse Transcription-Quantitative PCR (RT-qPCR)

An *A. fumigatus* inoculum reaching a total of 10^6^ conidia/mL was added to 100 mL of minimal medium broth and was grown for 18 h at 150 rpm and 37 °C. Voriconazole at 0.125 mg/L was added for 1h after overnight growth [44]. Mycelial samples were harvested using a funnel and miracloth (CalbiochemR, Merck Millipore, Madrid, Spain), blot dried, frozen with liquid nitrogen, and ground to powder. RNA was isolated by using an RNeasy plant minikit (Qiagen, Madrid, Spain) following the manufacturer’s instructions. DNA was eliminated using the DNA-free^TM^ Kit (Invitrogen, Madrid, Spain). RNA concentrations and quality were measured using NanoDropOne (Thermo Scientific, Madrid, Spain) and samples were conserved at −80 °C. Reverse transcription was carried out in a 20-μL reaction volume which contained 0.5 μg of an oligo(dT) 15 primer, 1 μL of reverse transcriptase from the ImProm-II Promega reverse transcription system (Promega, Madrid, Spain), and 1 μg of total *A. fumigatus* RNA. The reaction conditions were a first step of 5 min at 25 °C, then an hour at 42 °C and 15 min at 70 °C.

For transcription-level determination, a quantitative PCR (RT-qPCR) assay was performed using a CFX96 system (Bio-Rad, Madrid, Spain). The RT-qPCR conditions were 10 min at 95 °C and 40 cycles of 10 s at 95 °C, 5 s at 58 °C, and 30 s at 72 °C. The *cyp51A*, *cyp51B* and *hmg1* expression levels were quantified for each strain using the *A. fumigatus* β-tubulin gene (tub1, GenBank accession number AY048754) as a reference gene. All experiments complied with MIQE guidelines [45,46]. Primers used to amplify the cDNA from the *cyp51A*, *cyp51B* and *hmg1* genes are listed in Table S1. The primer set Tub5 (5’-TGACCCAGCAGATGTT-3’) and Tub6 (5’-GTTGTTGGGAATCCACTC-3’) was used for amplification of a fragment of the *A. fumigatus* β-tubulin gene. Bio-Rad RT-qPCR mixtures were set up with 10 μL of SensiMix SYBR-Hi carboxy-X-rhodamine (Bioline, Segovia, Spain), 0.6 μL MgCl_2_, 1.6 μL of each primer (10 μM) and 1 μg cDNA in a final volume of 20 μL. Each assay was repeated in triplicate with RNA from three different biological replicates. Each experiment included standard curves for the target genes (*cyp51A*, *cyp51B* and *hmg1*) and the reference gene (*tub1*). The efficiencies of RT-qPCR amplification of β-tubulin, *cyp51A*, *cyp51B* and *hmg1* were calculated from the slopes of the curves given by Bio-Rad CFX manager (version 2.0) software (Bio-Rad, Berkeley, California, USA), and the efficiency values were used to validate each experiment. Each RT-qPCR run included water as template for no template controls (NTC). Amplified products of expected 250–300 bp sizes were checked to verify the absence of unspecific RT-qPCR products or DNA contaminations through electrophoresis analysis. Fold changes in gene expression were calculated relative to the β-tubulin using the 2^-ΔΔCT^ threshold cycle (Cq) method [47]. Statistical analyses were performed with GraphPad Prism, version 5 Project (GraphPad Software, San Diego, CA, USA). The statistical significance of variances between fungal isolates was calculated by using a nonparametric Mann-Whitney t test. A *p*-value <0.01 was considered significant.

### 2.7. A. Fumigatus Cyp51B Protein three-dimensional (3D) Homology Modeling

Comparative models of Cyp51B proteins were performed. Cyp51B 3D theoretical models of the Cyp51B WT and the Cyp51B-G457S from *A. fumigatus* strains were made by automated homology modeling techniques using SWISS-MODEL [48,49]. The crystal structure of the 14-α sterol demethylase (Cyp51B) from *A. fumigatus* in complex with voriconazole (VCZ), deposited in the Protein Data Bank (PDB) under accession number 4UYM, was used as template. The Cyp51B N terminus (residues 1 to 49) constitute the membrane-spanning domain, which is not crystallized in the template protein.

## 3. Results

### 3.1. Antifungals Susceptibility Testing

In this study we analyze two *A. fumigatus* strains (CM8940 and CM9640) that were isolated, two years apart, from a patient suffering chronic pulmonary pathology with a long-term azole therapy. MICs to amphotericin B (AMB) and azoles itraconazole (ITC), voriconazole (VCZ), posaconazole (POS) and isavuconazole (ISA) were performed following the EUCAST methodology. Antifungal MIC values for *A. fumigatus* strains are detailed in Table 1. Strains were considered resistant with MICs over 2 mg/L to itraconazole (ITC) and voriconazole (VRC), over 0.25 mg/L to posaconazole (POS) and >1 mg/L to isavuconazole (ISA). Strain CM2580 was included as an *A. fumigatus* azole susceptible reference strain. Both *A. fumigatus* strains CM8940 and CM9640 were considered resistant to clinical azoles since their MICs were over the clinical breakpoints for these drugs [40]. Strain CM8940 had higher MIC values to ITC, POS and ISA but it was VCZ susceptible. However, strain CM9640 had elevated MICs to all clinical antifungals (>8) showing a higher azole MIC profile than strain CM8940. MICs for echinocandins were performed but no differences were observedt shown). Strain CM8693 (*A. lentulus*) showed the expected antifungal profile for *A. lentulus* [37] so this strain was not analyzed further (Table 1).

MICs were also performed for DMI drugs imazalil (IMZ), prochloraz (PRZ), metconazole (MET), tebuconazole (TEB), epoxiconazole (EPZ), bromuconazole (BRO) and difenoconazole (DIF) in our set of *A. fumigatus* strains (Table 2). Strain CM8693 (*A. lentulus*) was not included. Both strains CM8940 and CM9640 had higher MICs to all DMIs than WT reference strain CM2580 for both antifungal classes, imidazoles and triazoles. The MICs to DMIs were higher in strain CM9640 than in strain CM8940 for all drugs tested with MIC values of >32 mg/L for all triazole drugs.

### 3.2. Amplification and Sequence Analysis of Genes Involved in Azole Resistance

Amplification and sequencing of *cyp51A* and *cyp51B,* including their promoter regions, and *hmg1* in the two *A. fumigatus* strains detected specific mutations (Table 3). Strain CM8940 had a nucleotide substitution g161c (gGg/gCg) in *cyp51A,* which involves a G54A substitution in Cyp51A. Apart from a single c925t polymorphism in *cyp51B* not involving any amino acid substitution, no other changes were found in the sequences of *cyp51B*, its promoter or the *hmg1* gene. Strain CM9640 showed no changes in the coding sequence of *cyp51A* or its promoter. However, it had a substitution t1281c (Ttc/Ctc) in *cyp51B* that corresponds with a G457S amino acid substitution in the protein sequence; no changes were found in its promoter. Sequencing of *hmg1* showed a c2088t (Cat/Tat) substitution involving a F390L amino acid change. In addition, this isolate CM9640 harbored the same c925t polymorphism that CM8940 in *cyp51B* not involving any amino acid substitution (S244S).

### 3.3. Strain Genotyping

Both clinical *A. fumigatus* isolates, included in this study, had the same genotype (Table 3) and thus, were isogenic according to the TRESPERg typing assay, even though their MIC profiles and the amino acid substitutions found in resistance-associated genes were different.

### 3.4. Polymorphisms of Cyp51B and Hmg1 in A. fumigatus Azole Susceptible and Resistant Strains

As we have detected a variant in the *cyp51B* and *hmg1* genes in an *A. fumigatus* azole resistant strain, we decided to search for variants or mutations in Cyp51B and Hmg1 in a collection of 170 *A. fumigatus* genome sequences that had previously been sequenced in our laboratory or that we obtained from public databases [50]. All data used included information about antifungal susceptibility and azole resistance mechanisms. The analysis has revealed that *cyp51B* is a highly polymorphic gene, which can carry various synonymous nucleotide changes that are present in 0.5 to 75.2% of the strains analyzed (Table 4). However, only two non-synonymous mutations were found, the Q42L substitution present in 5.8% of the strains and the mutation D387E, which is harbored by 1.76% of the strains. Both mutations (Q42L and D387E) were found in azole-susceptible strains eliminating the possibility that they are directly involved in azole resistance.

We also used the same collection of 170 *A. fumigatus* genome sequences to search for SNPs in the *hmg1* gene concluding that *hmg1* is a highly polymorphic gene with abundant synonymous and non-synonymous polymorphisms. The polymorphisms that involved amino acid changes are summarized in Table 5. In total, 25% (43 of 170) of the strains sequenced had an amino acid substitution in Hmg1 and, among those, 44% of the strains (19 of 43) had a Cyp51A resistance mechanism in combination. In addition, some mutations appeared in susceptible strains excluding their involvement in resistance.

### 3.5. Cyp51A, Cyp51B and Hmg1 Expression Analysis with and Without Azole Induction

**Figure 1:**
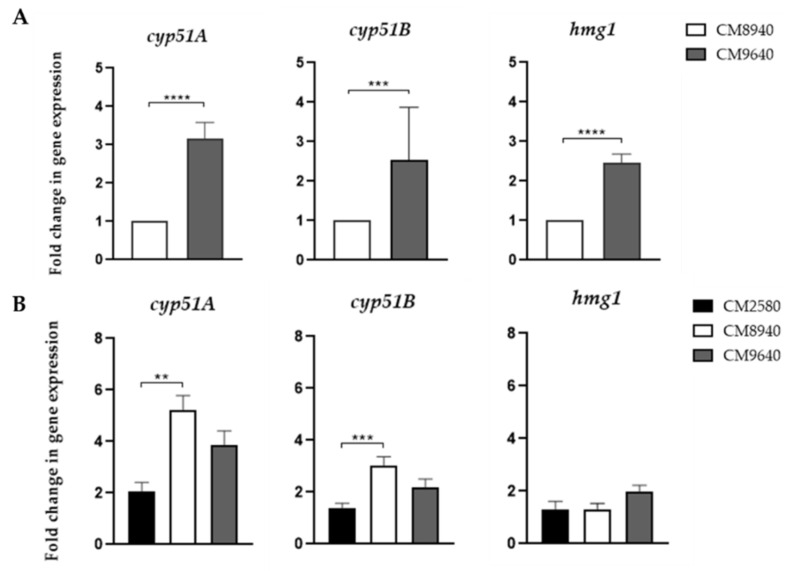
(**A**) Fold changes in expression of *cyp51A*, *cyp51B* and *hmg1* for CM8940 and CM9640 isolates. (**B**) Fold changes in expression of *cyp51A*, *cyp51B* and *hmg1* with and without VCZ induction for CM2580, CM8940 and CM9640 strains. Mann-Whitney t test was performed to statistical significance. P values under 0.01 were considered significant. ** *p*<0.001, *** *p*<0.0001, **** *p*<0.00001.

Expression analysis of *cyp51A*, *cyp51B* and *hmg1* genes were carried out by RT-qPCR on the *A. fumigatus* strains CM8940 and CM9640 (Figure 1A). We calculated relative expression of each gene to the CM8940 strain (isogenic to CM9640). In standard conditions expression of all the genes analyzed was significantly higher for the strain CM9640. We then analyzed the effect when the strains were incubated for one hour in the presence of voriconazole. We compared gene expression for a wild type susceptible strain (CM2580), CM8940 and CM9640 strains grown in the absence and presence of voriconazole (Figure 1B). We found a significant increase in gene expression of *cyp51A* and *cyp51B* when the strain CM8940 was treated with voriconazole compared to the susceptible strain. The strain CM9640 showed a slight, but not significant, expression increase of all three genes in the presence of voriconazole.

### 3.6. Cyp51B Protein 3D Homology Modeling

**Figure 2:**
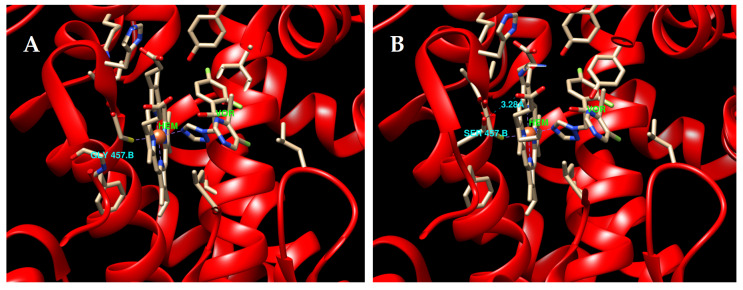
Crystal structure of 14-α sterol demethylase (Cyp51B) from *A. fumigatus* in complex with voriconazole. (**A**) Structure of WT Cyp51B with the amino acid glycine in position 457. (**B**) Structure of Cyp51B with the substitution of the glycine for a serine in the position 457 of the protein. Hydrogen bonds between serine 457 and heme group are shown as cyan dashes and its length is 3.28 Å. The carbon atoms of the voriconazole and the heme (stick representations) are both in gray and labeled in green (VOR and HEM). The iron is depicted as an orange sphere. The protein backbone is colored in red.

In order to explore potential structural differences between *A. fumigatus* strains with wild-type Cyp51B and Cyp51B-G457S and to address the relationship between this mutation and azole resistance, two Cyp51B 3D homology models were constructed (Figure 2). As expected, both homology model structures were almost identical owing to their nearly matching amino acid sequences. The 3D conformational structure was only different in position 457, due to the change of glycine (G) for serine (S). The amino acid in position 457 is located in the heme-binding domain [51] and the substitution of the amino acid residue in this position likely affects the interaction with the heme group, since the distance of residue chain and the heme group is reduced (Figure 2).

## 4. Discussion

Over the past two decades, numerous studies have investigated and identified mechanisms of azole resistance in *A. fumigatus* [9]. Most of them refer to alterations in *cyp51A* structure, or its expression level, as the main mechanisms of resistance in this fungus, which causes treatment failure in aspergillosis patients infected with azole resistant strains [52]. Two ways of azole resistance development have been reported: An environmental route, based on the use of fungicides to protect crops, which involves tandem repeat insertions in the promoter of the *cyp51A* gene in combination, or not, with point mutations in the coding sequence [9,10]; and a medical route as a result of long-term azole therapies, inducing the development of resistance mechanisms mainly consisting in point mutations in the *cyp51A* gene (G54, G138, M220, P216 and G448) [53,54]. However, azole resistant strains lacking *cyp51A*-related resistance mechanisms have always been left behind due to the lower frequency of these isolates [9]. More recently, the incidence of these *cyp51A* wild-type resistant isolates is increasing and, with it, the number of studies investigating other azole resistance mechanisms Cyp51A-independent [11,13,14,15,16,17,18,19,20,21,22,24,25,55].

In this study, we included two *A. fumigatus* clinical strains that were isolated from a patient that had been under prolonged azole treatment. Both strains, CM8940 and CM9640, isolated two years apart, were isogenic. However, each strain had different gene mutations and a different susceptibility profile to clinical and environmental azole drugs. In the absence of clinical data, we hypothesize that this different azole profile is likely related to the azole therapy that the patient was receiving, with different selection of resistance mechanisms, depending on the azole used. However, we do not exclude the possibility that both strains may be coexisting in time.

Cyp51s are 14-α sterol demethylase enzymes that belong to the cytochrome P450 family [56]. They are rate-limiting enzymes in the ergosterol biosynthesis pathway where they mediate the conversion of eburicol into 4,4-dimethylcholesta-8,14,24-trienol [6]. *A. fumigatus* has two different but related enzymes that mediate this step, Cyp51A and Cyp51B [5], both of which are successfully inhibited by azole drugs, suggesting that both enzymes can play a role in azole resistance [7,8]. 

The strain CM8940 has a G54A substitution in Cyp51A and showed high MICs to ITC and POS. This mutation had been previously described and associated with azole resistance [57]. After observing the elevated azoles MIC profile of strain CM9640, the *cyp51A* gene and its promoter were amplified and sequenced. However, we did not find any modification related to the *cyp51A* gene. In order to further investigate the resistance mechanism present in this strain, we looked for mutations in Cyp51B and also in Hmg1, an enzyme involved in the ergosterol biosynthetic pathway that has recently been implicated in azole resistance [13,14,15,16,17,18,19]. Interestingly, we found that the strain CM9640 has two amino acid mutations: a G457S in Cyp51B and a F390L in Hmg1.

**Figure 3:**
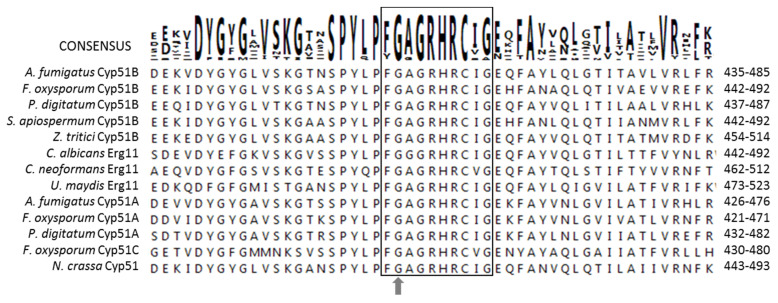
Multialignement of 50 amino acid residues of Cyp51/Erg11 sequences from different fungal species: *Aspergillus fumigatus* (AfCyp51A; GenBank accession no. AAK73659; and AfCyp51B; GenBank accession no. AAK73660), *Candida albicans* (CaErg11; GenBank accession no. AAF00598), *Cryptococcus neoformans* (CnCyp51; GenBank accession no. AAF35366), *Fusarium oxysporum* (FoCyp51A; GenBank accession no. SCO89707; FoCyp51B; GenBank accession no. XP_031042981; and FoCyp51C accession no. XP_031031054.1), *Penicillium digitatum* (Pd Cyp51A; GenBank accession no. XM_014676686.1; and Pd Cyp51B; GenBank accession no. HQ724322.1), *Neurospora crassa* (Cyp51; GenBank accession no. XM_009858357.1), *Scedosporium apiospermum* (Cyp51B; GenBank accession no. MH120957.1), *Ustilago maydis* (Erg11; GenBank accession no. CAA88176) and *Zymoseptoria tritici* (Cyp51B, GenBank accession no. EU418063.1). Numbers in the right indicate amino acid positions of the proteins, the heme binding domain is indicated by a square and the location of the G457 amino acid position in *A. fumigatus* Cyp51B and its homologues is indicated by an arrow.

Multiple sequence alignment of *A. fumigatus* Cyp51A, Cyp51B and other Cyp51s protein sequences from different fungal species (Figure 3) showed that the glycine in position 457 in Cyp51B corresponds to the glycine in position 448 of *A. fumigatus* Cyp51A. In Cyp51A, substitution of this glycine for a serine (G448S) has been described and proved to confer azole resistance in vitro and in vivo [58,59,60,61,62]. Indeed, G448S is one of the main Cyp51A azole resistance mechanisms, which has been seen to confer resistance to voriconazole and isavuconazole, and slightly elevated MICs to itraconazole and posaconazol [58,62,63,64,65]. All the Cyp51A-G448S isolates described to date have been obtained from patients that were previously treated with voriconazole [58,62,63,64,65]. The alignment of a variety of Cyp51/Erg11 fungal proteins showed that the heme binding domain, which includes the Cyp51B-G457 and Cyp51A-G448 amino acid, is conserved among all of them (Figure 3). The azole MICs observed in strain CM9640 (Cyp51B-G457S) are higher than the MICs of those strains harboring the G448S modification in Cyp51A, which is known to confer resistance only to voriconazole and isavuconazole. This suggests a possible concomitance with other azole resistance mechanisms.

The Cyp51 proteins are competitively inhibited by azoles, and are present in all biological kingdoms [66]. Among them, the primary amino acid sequence identity usually ranges between 20–30% [67]. However, all Cyp51s show high conservation of specific amino acids constituting conserved domains along the phylogeny, indicating the key roles that they may play in the structure/function of this monooxygenase. The 3D protein structural models of both Cyp51Bs (wild-type and G457S) (Figure 2) show that the glycine in position 457 is located very close to the catalytic heme binding domain, which is involved in the interaction of the substrate/drug with the enzyme and is completely covered inside the protein. This domain is highly conserved among eukaryotic and bacterial Cyp51s [51,68]. The glycine at position 457 in Cyp51B is close to the heme group but is not within the 3Å environment of this group. In wild-type Cyp51B, the G457 cannot interact with azole drugs directly; however, this position might be important for the conformation of the heme environment. When the glycine is substituted for a serine, the nucleophilic character of this amino acid and the fact that the side chain of the serine is larger than the glycine may be affecting the environment of the heme group (Figure 2). Changing glycine for other amino acid residues would be expected to decrease the flexibility required for interdomain conformational changes over inhibitor or substrate binding. As it happens with the replacement of the glycine for a serine in position 448 of *A. fumigatus* Cyp51A, the change of the amino acid blocks the access of azoles to the heme group and, thus, it reduces the ability of azoles to bind effectively inhibiting their function. The effects of this substitution have been previously reported in the same position in *A. fumigatus* Cyp51A (G448) [69,70] and in Erg11 in yeasts, including *Candida* spp. (G464D/S) and *Cryptococcus neoformans* (G484S), all of them associated with azole resistance [71,72,73,74,75]. Mutations at the same corresponding amino acid position have also been found in Cyp51s proteins of plant pathogen fungi, where this substitution has been linked to resistance to demethylation inhibitors (DMIs) [36,76,77,78]. In *Penicilium digitatum*, *Ustilago maydis, Pyrenopeziza brassicae* or *Scedosporium* spp. substitutions at the same position in their Cyp51B homologues have been described related with resistance to different DMIs [27,36,76,77].

In *A. fumigatus, hmg1* encodes an HMG-CoA reductase (HMGR), an enzyme that participates in one of the first steps of the ergosterol pathway catalyzing the conversion of 3-hydroxy-3-methylglutaryl-CoA in mevalonic acid [79]. Mutations in Hmg1, more specifically in the sterol-sensing domain (SSD) of the protein, are one of the recently reported azole resistance mechanisms in strains with a wild-type *cyp51A*, although the underlying reason for these mechanisms has not been elucidated yet [13,14,15,16,17,18,19,80]. In addition, most of the strains that had resistant MICs profiles and *hmg1* mutations also had modifications in *cyp51A* in combination [13,14,16,17,18,19]. Curiously, some of them harbored the G448S mutation in Cyp51A as the resistance mechanism found [13]. Strain CM9640 also harbored a modification in the SSD of Hmg1 (F390L) in addition to the Cyp51B (G457S) that could explain its panazole resistant phenotype.

The search for the Hmg1 polymorphisms in the genome of 170 *A. fumigatus* strains showed that *hmg1* is a highly polymorphic gene and several alterations in its sequence were found in azole susceptible as well as in azole-resistant strains (Table 5). Previous studies propose that mutations in *hmg1* could appear as a general adaptation mechanism of fungi under triazole pressure, only conferring moderate increments in the azole MICs and facilitating the development of *cyp51A* modifications [14,81,82]. Nevertheless, further analyses need to be performed in order to understand the contribution of the detected gene modifications in the *A. fumigatus* CM9640 azole resistance profile. In particular, it will be important to generate mutant strains carrying individual mutations in Cyp51B or Hmg1, in order to elucidate the specific role of each of them in *A. fumigatus* azole resistance.

Cyp51B, as well as Cyp51A, has proven to be a functional enzyme that is expressed in regular conditions and which is efficiently inhibited by azoles [5,48]. The over-expression of *cyp51B* has been observed in in vitro analysis and its role in azole resistance has been proposed opening the possibility of a novel azole drug resistance mechanism [11]. We performed expression analysis of genes *cyp51A*, *cyp51B* and *hmg1* with, and without, VCZ stimulation. Our results show that strain CM9640, harboring mutations in Cyp51B and Hmg1, had higher levels of expression of the three genes studied than strain CM8940 in standard conditions. These data suggest that the overexpression of the three genes can be due to the substitution in *cyp51B*, *hmg1* or to both together. Mutation in *hmg1* could be inducing a higher expression of *cyp51A*, which is in agreement with the previous finding of Wu et al. [16] who described an azole resistant *hmg1*-mutated strain with overexpression of *cyp51A*. However, Rybak et al. [14] did not find any overexpression of *cyp51A* in *hmg1*-mutated strains, which underlines that the involvement of *hmg1* mutations in *cyp51A* expression requires further exploration. 

In addition, the mutation in *cyp51B*, which is located in the heme binding domain of the protein, does not only affect its binding to azole drugs, but also with its substrate, which reduces the activity of the protein, and therefore suggests that the increase of expression of both genes is a compensatory mechanism for this reduced Cyp51B enzyme activity [8]. The increase of expression of both *cyp51*-related genes and its implication in azole resistance has been already described by Brillowska-Dabrowska et al. [83] suggesting the possibility of other mechanisms being involved in the modulation of the expression of these genes. 

Finally the higher expression of *cyp51A* and cyp51B in strain CM8940 in the presence of voriconazole is unexpected since Cyp51A G54 mutations in have never been related to differences in *cyp51A* gene expression, although more likely because it was never investigated. However, it is worth mentioning that the higher expression levels of *cyp51A* and *cyp51B* in this strain could be related to the high MICs to VCZ and ISV, which is not the characteristic azole profile of strains with G54 mutations in Cyp51A (resistant only to ITC and POS) [9].

## 5. Conclusions

In conclusion, we describe a possible new mechanism of azole resistance in *A. fumigatus* related to a modification in Cyp51B (G457S) in combination with a modification in Hmg1 (F390L). Until now, mutations in Cyp51B were rarely investigated assuming that Cyp51A was the only enzyme involved in resistance. However, the results obtained in this work highlight the relevance of investigating alterations in Cyp51B alone, and in combination with other enzymes of the ergosterol biosynthesis pathway, as a possible mechanism of azole resistance, especially in patients under prolonged azole treatment. In order to undoubtedly link alterations in Cyp51B to azole resistance and to elucidate the individual contributions of each mutation to the observed azole resistant phenotype, single and combined mutant strains are currently under construction in our laboratory.

## Figures and Tables

**Table 1:** Minimal inhibitory concentrations (MICs) for *A. fumigatus* and *A. lentulus* isolates against antifungal drugs (AMB: Amphotericin B, ITC: itraconazole, VCZ: voriconazole, POS: posaconazole, ISA: isavuconazole).

Strains	Species Identification	Collection Date	MICs (mg/L)				
			AMB	ITC	VCZ	POS	ISA
CM2580	*A. fumigatus*	10/06/20	0.5	0.25	0.5	0.06	0.5
CM8693	*A. lentulus*	23/09/16	2	0.5	4	0.25	2
CM8940	*A. fumigatus*	24/05/17	2–4	>8	0.5–2	1–4	8
CM9640	*A. fumigatus*	13/06/19	1–2	>8	>8	>8	>8

**Table 2:** Minimal inhibitory concentrations (MICs) in mg/L for *A. fumigatus* isolates against 14-α sterol demethylation inhibitors (DMIs) (IMZ: imazalil, PRZ: prochloraz, MET: metconazole, TEB: tebuconazole, EPZ: epoxiconazole, BRO: bromuconazole, DIF: difenoconazole).

*A. fumigatus* Strains	Imidazoles		Triazoles				
	IMZ	PRZ	MET	TEB	EPZ	BRO	DIF
CM2580	0.125–0.5	0.125–0.5	0.125–0.5	1–2	1–4	0.5–2	0.5–2
CM8940	0.5–1	0.5–1	1–2	8–16	8–16	>32	2–8
CM9640	4–8	8	>32	>32	>32	>32	>32

**Table 3:** Amino acid substitutions in Cyp51A, Cyp51B and Hmg1 and TRESPERg genotype in the two clinical azole-resistant *A. fumigatus* strains.

Strains	Amino Acid Substitutions			TRESPERg				Genotype
	Cyp51A	Cyp51B	Hmg1	CSP	Mp2	CFEM	Erg4B	
CM8940	**G54A**	WT	WT	4.1	1.3	8.1	7	t04Am1.3c08A.e07
CM9640	WT	**G457S**	**F390L**	4.1	1.3	8.1	7	t04Am1.3c08A.e07

**Table 4:** Analysis of the *cyp51B* polymorphisms and Cyp51B amino acid substitutions found in our set of 170 *A. fumigatus* clinical strains. Bold letters indicate amino acid changes.

Nucleotide Position (cDNA)	Codon Change	Amino Acid Change	Number of Strains	Percentage (%)
105	tcT/tcC	S35S	31	18.2
**125**	**cAg/cTg**	**Q42L**	**10**	**5.8**
468	tcT/tcA	S156S	1	0.5
561	ttC/ttT	F187F	2	1.17
564	gaT/gaC	D188D	1	0.5
822	tcC/tcT	S274S	18	10.0
**1161**	**gaT/gaA**	**D387E**	**3**	**1.76**
1182	ccT/ccG	P394P	128	75.2
1392	atT/atA	I464I	17	10

**Table 5:** Analysis of the Hmg1 Amino Acid Substitutions Found in Our Set of 170 *A. Fumigatus* Clinical Strains.

Nucleotide Position (cDNA)	Codon Change	Amino Acid Change	Nº of Strains	Azole S	Azole R	Cyp51A R Mechanisms	Percentage (%)
313	Gag/Aag	E105K	10	1	9	TR34/L98H, G138C	5.8
634	Tcg/Ccg	S212P	1	1	0	--	0.5
919	Ggc/Agc	G307S	4	0	4	G138C	2.4
955	Aag/Cag	K319Q	1	0	1	M220I/V101F	0.5
1102	Tac/Cac	Y368H	1	1	0	P216L	0.5
1235	aTc/aCc	I412T	2	2	0	--	1.17
1454	tTt/tCt	F485S	1	0	1	P216L	0.5
1621	Agc/Ggc	S541G	8	4	4	M220T, G54E, TR34/L98H	4.7
1690	Tac/Cac	Y564H	15	15	0	--	8.8

S: susceptible; R: resistant/resistance.

## Supplementary Materials

The following are available online at https://www.mdpi.com/2309-608X/6/4/315/s1, Table S1: Primers used for amplifying, sequencing and qPCR of *cyp51A*, *cyp51B*, their promoters and *hmg1* gene.
