# Supplementary material for: A Cyp51B Mutation Contributes to Azole Resistance in Aspergillus fumigatus
Source: J Fungi (Basel). 2020 Nov 26;6(4):315. doi: 10.3390/jof6040315 (PMC7712412; doi:10.3390/jof6040315)
Supplement: Supplementary file 1 [file jof-06-00315-s001.pdf]

**Supplementary Table 1:** Primers used for amplifying, sequencing and qPCR of *cyp51A*, *cyp51B*, their promoters and *hmg1* gene.

| Gene | Primer name | Sequence (5′-3′) | Application | Origin |
|---|---|---|---|---|
| ***cyp51A* promoter** | A5R | TCTCTGCACGCAAAGAAGAAC | PCR and sequencing | [5] |
| | A7F | TCATATGTTGCTCAGCGG | | |
| ***cyp51A*** | P450.1F | ATGGTGCCGATGCTATGG | PCR and sequencing | |
| | P450.2R | CTGTCTCACTTGGATGTG | | |
| | A1F* | CTTCTTTGCGTGCAGAGA | Sequencing | |
| | A3F | TAGTCCATTGACGACCCC | | |
| | A4F | CAGACATGATATGGAACC | | |
| | A10R* | ATTGCCGCAGAGATGTCC | | |
| | Afer12R | GGGAGGAATCATGTAAGGGG | | |
| | CypA1F | CTTACGGCCTACATGGCC | | |
| | CypA2R | TTCGACCGCTTCTCCCAG | | |
| ***cyp51B* promoter** | B5R | TTGCGCGGCAGTCGAAAAAGAAC | PCR and sequencing | |
| | B7F | ACTAGGGTCAGTTAGTCC | | |
| ***cyp51B*** | P450.3F | ATGGGTCTCATCGCGTTC | PCR and sequencing | |
| | P450.4R | TCAGGCTTTGGTAGCGG | | |
| | B1.F* | CTTTTTCGACTGCCGCGC | Sequencing | |
| | B2.R* | AGGCGTAGTGAGTGGAGA | | |
| | B3.F | TCTCCACTCACTACGCCT | | |
| | B5.R | TTGCGCGGCAGTCGAAAAAGAAC | | |
| | B6.F | CATGGCTGTGGATGGTACTTC | | |
| | B8.F | CAGTGAAGAATCCCATGG | | |
| | Afer7.R | CCARCGRTGNGGRTCCCA | | |
| ***hmg1*** | P3F | GCTACCTCTCTGATTACAAGG | PCR and sequencing | This study |
| | P4R | GCCAACCAGAGCTTGATAGC | | |
| | P1F | TGCTATCAAGCTCTGGTTGG | | |
| | P2R | CAGAGGCCAATCATTACTGG | | |
| | P5F | GAAGATGGTATCACCCATCGG | | |
| | P6R | CTCGCTTCTCTTTCAAGAAGG | | |
| | P7F | CACTCCCAAGAGGAGTTTAG | | |
| | P8R | CACTCAGTCGCCATAACGTGG | | |
| | P9F | GGCACTTATCTTTACATCCGG | | |
| | P10R | CGAGGAGGACATCGTCAATC | | |
| | RT5R* | TTCAACGCTCACGCATCGAC | RT-qPCR | |
| | RT6R* | ACCTAGCATATCAAGCATGGC | | |

Primers marked with an * were used for RT-qPCR assays.
